# Age assessment services in public dental health facilities in Kenya: burden and sources of referral (a cross-sectional study)

**DOI:** 10.11604/pamj.2023.45.125.37365

**Published:** 2023-07-14

**Authors:** Eunice Njeri Kihara, Simon Muturi Karanja, Peter Wanzala, Evelyn Gaceri Wagaiyu

**Affiliations:** 1 School of Public Health, Jomo Kenyatta University of Science and Technology, Nairobi, Kenya,; 2 Centre for Public Health Research, Kenya Medical Research Institute, Nairobi, Kenya,; 3 Department of Periodontology/Community and Preventive Dentistry, School of Dental Sciences, University of Nairobi, Nairobi, Kenya

**Keywords:** Dental age, assessment, estimation, forensic odontology, dentistry

## Abstract

**Introduction:**

the need for age assessment remains relevant due to unregistered births or lack of identification documents as a result of political and socioeconomic conflicts. Dentists play a significant role in age assessment. In order to establish the need for capacity building and collaboration, the study set out to determine the proportion of dental patients referred for age assessment and their sources of referral.

**Methods:**

a cross-sectional study, based on 5-year records (2014-2018) of dental patients and a selected sample of 316 patients who visited for age assessment in 2019-2020 in the same hospitals. The study centers were 8 county hospitals. Variables included gender, dental visits, health facility, and sources of referral. One-way Analysis of Variance (ANOVA) and Fisher´s Exact test were used to identify a significant association between variables.

**Results:**

from the records, 12,738 (5.7%) patients sought age assessment services. Females 6,410 (50.3%) and males 6,328 (49.7%) were nearly equal. There was a statistically significant difference in the average number of patients who required age assessment services from different facilities, p=0.000. The selected sample comprised of 186 (58.9%) and 130 (41.1%) males and females respectively. Sources of referral included; court of law (267, 84.5%), prior to acquiring identification documents (47, 14.9%), and others (2, 0.6%).

**Conclusion:**

an average of 5% of dental patients seek age assessment services signifying the need for sustained capacity building so as to ensure competent service delivery. The majority of the sampled patients were referrals from the court of law. Further research on how the dental fraternity can collaborate with the Judiciary to ensure justice in age disputes is recommended.

## Introduction

Birth and civil registration allow one to officially exist and benefit from a myriad of legal and social services as well as providing a sense of belonging. Nevertheless, due to varying political, social, and economic challenges, there exist individuals who have never been registered. Lack of registration highly violates human rights. According to United Nations Children's Fund, globally, 237 million children aged 5 years and below do not possess birth certificates and about a third of them (66 million, 28%) are found in East and Southern Africa [1,2]. In Kenya, 23% of those under 5 years old were not registered by 2014 [3]. Hence, age assessment remains a necessity [4]. Documentary evidence comes in handy in a court of law when a judge has to distinguish between the crimes of rape and defilement whose victims are adults and children respectively [5,6]. Age also needs to be confirmed when a decision has to be made on whether to commit one to juvenile or adult jail [7]. Hence, mistakes lead to unwarranted penalties.

Difficult situations have resulted in a global rise in refugees, internally displaced persons, and asylum seekers. This is due to terrorism; poor socioeconomic conditions; natural disasters like drought, floods, landslides, and storms; political violence associated with national elections; inter-communal conflicts, and eviction of illegal squatters from forests or development-induced displacement [8]. In these situations, individuals may lose legal identification documents necessitating age assessment (AA). Other individuals who may lack identification documents include trafficked children, street families, as well as some children under foster care. The 2020 global report on human trafficking indicated that the majority (2,833, 59%) of victims from sub-Saharan Africa were children [9]. United Nations High Commissioner for Refugees and other non-governmental organizations endeavor to protect refugees and other lonely children by ensuring prompt access to legal support, education, and other basic needs once the age is established [10,11]. In Kenya, an unaccompanied child whose age is determined to be 8 years and below has the benefit of automatically acquiring the status of a Kenyan citizen [7].

Age can be established through documentary evidence. However, when sufficient and reliable evidence is not available, a multidisciplinary team of healthcare professionals plays a crucial role in assessing certain psychological, physical, and developmental features that indicate a probable age. The process of AA is challenging and the estimated age needs to be interpreted cautiously in order to protect human rights. Therefore, there is a need for continued collaboration between those who refer patients for age determination and the assessors. In particular, dentists play a significant role in providing expert evidence in AA matters in Kenya. Nonetheless, in Kenya, there is paucity of forensic dentists, and forensic age assessment techniques are minimally taught. In dental training institutions, age assessment is mostly done for clinical purposes of assessing growth before treatment planning. The aim of the study was to establish the number of patients who sought AA in public dental facilities within a 5-year period as well as interviewing a selected sample of age assessment patients so as to establish their sources/reasons for referral. This provides baseline information that justifies the need to reevaluate the training on forensic dentistry for dental students and continuous development programs for relevant stakeholders.

## Methods

**Study design and setting:** this was a cross-sectional study involving two study populations selected from 8 dental facilities located at level five county hospitals. Hospitals were selected through stratified and simple random methods. The sampling frame pooled 41 level 5 county hospitals which were stratified according to their location in the former eight administrative provinces of Kenya. One hospital was selected from each category through computer-generated random numbers using a Microsoft Excel spreadsheet.

**Study population 1 and data collection method from 5-year records:** this involved a census of dental patients who sought routine dental procedures and AA services over a five-year period (January 2014-December 2018). Complete enumeration was done to determine the proportion of female and male dental patients who sought AA services. A data collection form was used to compile yearly data. Outcome variables included gender and annual proportions of patients visiting the selected facilities.

**Study population 2 and data collection method from a selected group of patients who sought age assessment services:** only a convenience sample of patients who visited the dental facilities for AA during the study period (November 2019-December 2020) were selected. This was done to avoid missing data which was not routinely documented in the patient´s clinical records. The sample size was determined to be 320 through Cochrane´s formula. Due to the paucity of information from previous studies, a prevalence of 50% was assumed. The confidence level was set at 95%. A correction factor was applied since a finite population was expected to seek age estimation services over the study period. Outcome variables included gender, estimated age, and sources of referral for AA.

**Data analysis:** descriptive and inferential statistics were done using the Statistical Package for the Social Sciences (SPSS) version 26. Data were classified according to gender, annual attendance, and provincial location of the hospitals. Data were described according to frequencies, mean, standard deviation, median, range, and yearly trends. Levene´s test was used to assess the equality of variances of the regional and annual means of dental and AA visits. One-way ANOVA or Kruskal-Wallis H test of significance were applied where population variance was equal or unequal respectively. Therefore, one-way ANOVA was used to test the statistical significance of regional and annual mean differences in general dental visits. Where significant results were found, Tukey´s honestly significant difference (HSD) post hoc test was done. Kruskal-Wallis H test was used to test the regional differences in the average number of patients visiting for AA. Fisher´s Exact test was used to determine if there was a significant association between the location of the health facility and sources of referral for AA. The level of significance (p-value) was set at <0.05.

**Ethical considerations:** authority to conduct the study was approved by the Kenyatta National Hospital/University of Nairobi (KNH/UON)-Ethics and Research Committee (ERC) (P104/02/2019). Authority was also granted by the Ethical Committees of the participating health facilities and the National Commission for Science, Technology and Innovation (NACOSTI/P/20/1105 and NACOSTI/P/19/1105). Data were collected from patients who consented/assented to undergo the AA process. The data was coded and stored anonymously.

## Results

**Figure 1:**
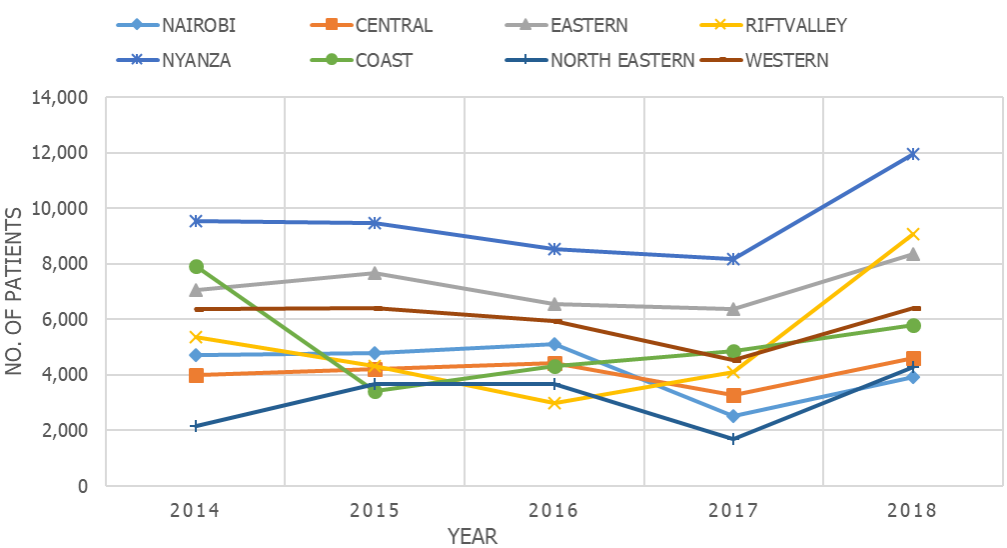
five-year trend in overall dental visits in selected hospitals from different regions in Kenya

**Data from 5-year dental records:** from the 8 study centers, 222,456 patients attended the dental facilities from 2014 -2018 (Table 1). The mean from the 8 study centers was 5561.4 ± 2276.34 with a range of 1,703 - 11,949 patients. Among them, 95,663 (43.00%) males and 126,793 (57.00%) females at a ratio of 1: 1.3. One-way ANOVA revealed significant regional differences in the mean number of patients attending the dental facilities in 5 years (F (7, 32) = (11.416), p = 0.000). The majority of dental patients were seen in the facility located in Nyanza (47,665, 21.42%) while the least were in the North Eastern region facility (15,486, 6.96%) (Table 1). Tukey´s HSD test revealed that there were significant (p ≤000) differences when the Nyanza facility was compared with all the facilities except the one from the Eastern region (p=0.146). The percentage annual change was negative (-7.1%, -6%, -16.6% from 2014 to 2017) followed by a 34% increase in 2018. However, the annual differences were not statistically significant (F (4, 35) = (1.187), p=0.334) (Figure 1).

**Table 1:** total number of dental and age assessment patients according to gender and geographical location

Provincial region	Dental visits				Visits for age assessment			
	Female (%)	Male (%)	Total (%)	Mean/ year	Female (%)	Male (%)	Total (%)	Mean/ year
Central	12,494(60.9)	8,014(39.1)	20,508(9.2)	4101.6	113(35.9)	202(64.1)	315(2.5)	63.0
Coast	14,316(54.5)	11,956(45.5)	26,272(11.8)	5254.4	725(54.8)	598(45.2)	1,323(10.4)	264.6
Eastern	21,821(60.6)	14,171(39.3)	35,992(16.2)	7198.4	469(47.2)	530(52.8)	999(7.8)	199.8
Nairobi	11,382(54.1)	9,653(45.9)	21,035(9.5)	4207.0	1,087(53.0)	962(47.0)	2,049(16.1)	409.8
North Eastern	8,299(53.6)	7,187(46.4)	15,486(7.0)	3097.2	3,015(50.7)	2,929(49.3)	5,944(46.7)	1,188.8
Nyanza	26,259(55.1)	21,396(44.9)	47,655(21.4)	9531.0	183(40.5)	269(59.5)	452(3.6)	90.4
Rift Valley	15,048(58.2)	10,799(41.7)	25,847(11.6)	5169.4	722(60.0)	482(40.0)	1,204(9.5)	240.8
Western	17,174(57.9)	12,487(42.1)	29,661(13.3)	5932.2	96(21.2)	356(78.8)	452(3.6)	90.4
Total	126,793(57.0)	95,663(43.0)	222,456(100)	5561.4	6,410(50.3)	6,328(49.7)	12,738(100)	318.45

A total of 12,738 (5.73%) dental patients visited for AA between 2014-2018 among the dental patients. The mean for the 8 study centers over the 5-year period was 318.45 ± 394.53 with a range of 43 - 1,797 patients. Females (6,410, 50.32%) and males (6,328, 49.68%) were nearly equal. Notably, about half of the patients requiring age estimation were recorded in the North Eastern health facility (5,944, 46.66%) while the lowest cases were found in the Central region health facility (315, 2.47%). Levene´s test of homogeneity of variances was statistically significant F (7,32)=11.222, p≤000. Hence, Kruskal-Wallis H test was applied which revealed statistically significant regional differences in the average number of patients who sought AA services in 5 years, χ^2^(7)=30.605, p≤0.000 (Table 1).

**Figure 2:**
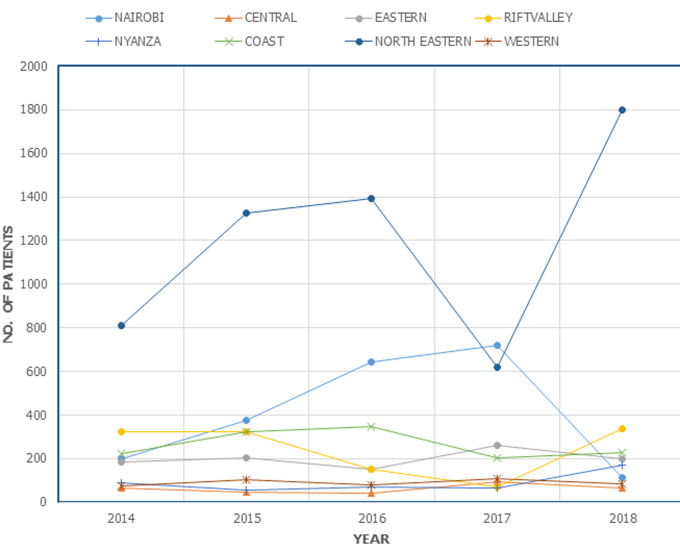
five-year trend in age assessment visits in selected hospitals from different regions in Kenya

Overall annual percentage change in age assessment visits from 2014 to 2018 was 29%, 4%, -35%, and 29% respectively. However, one-way ANOVA did not reveal any significant mean annual differences (F (4, 35) = (0.157), p=0.959) (Figure 2). Compared to other dental procedures, AA accounted for 0.95 - 9.74% in seven (7) facilities while the North Eastern facility had slightly over a third (38.38%) of AA patients.

**Data from a select population that sought age assessment services:** data were collected from a sample of 316 patients who visited for AA. The ratio of female to male patients varied from 1: 1.02 to 1: 1.8 in 7 centers, however, one health facility selected from Rift Valley had more females than males (1: 3.7). The mean age was 19.4 ± 12.3 years. A majority (267,84.5%) of the sampled patients were referred by the law courts (Table 2). The rest of the patients needed age assessment prior to acquiring identification documents (47,14.9%), school admission (1,0.3%), and referral from a private caregiver (1,0.3%). Identification documents included national identity cards (28,8.86%), passport/travel documents (12,3.8%), and birth certificates (7,2.22%). Among those who required identification documents were mainly from the North Eastern region (27,57.45%), Coast (15,31.91%), and Nairobi (5,10.64%). A Fisher´s Exact test showed significant association between location of the health facility and sources for referral (p ≤0.000).

**Table 2:** sources of referral for age assessment according to the location of the health facility

Provincial region	Court of law n (%)	Others n (%)	Total	Chi-square test (X^2^)
Central	40(100.0)	0	40	p=0.000
Coast	25(62.5)	15(37.5)	40	
Eastern	38(100.0)	0	38	
Nairobi	33(82.5)	7(17.5)	40	
North Eastern	13(32.5)	27(67.5)	40	
Nyanza	40(100.0)	0	40	
Rift Valley	40(100.0)	0	40	
Western	38(100.0)	0	38	
Total	267(84.5)	49(15.5)	316(100)	

## Discussion

**Statement of principal findings:** the study found an overall positive trend in average annual visits for routine dental and AA services in level 5 health institutions. The burden for dental care and AA procedures was influenced by the location of the health facility. Age assessment procedures were equally sought for by both genders. In addition, it was observed that the court of law was the commonest source of referral for AA.

**Strengths and weaknesses of the study:** the study was based on eight health facilities spread across all the 8 provincial regions of Kenya. The sampled facilities were major teaching and referral health institutions which were classified as level 5 hospitals. The data was from a 5-year record of dental patients from each institution. Data on sources of referral was collected prospectively from patients who were visiting the dental clinics during the data collection period of 2019-2020. Therefore, recall bias or missing data which would have acted as confounders were avoided. The five-year AA records did not have information on sources of referral, hence, it was impossible to analyse the five-year trend.

**Strengths and weaknesses in relation to other studies:** studies on the burden of AA and sources of referral are hardly published. Nevertheless, we found few studies which were based on single institutions while our study involved 8 institutions, hence, our sample size was larger. The current data was collected from public dental facilities while other published studies were based on institutions specifically dealing with legal and forensic matters [12,13]. Hence, detailed records of patients with disputed ages were available. Our study did not find consistent and comprehensive clinical records on previous AA procedures making it difficult to assess additional variables.

**Discussion of important differences in results:** a wide variation in the number of patients who sought AA services was observed among the selected hospitals. The differences could be influenced by population density, and regional disparities in social, political, and security conflicts resulting in lack of legally approved identification and birth documents [14,15]. Other differences could be due to proximity to the law courts [16] which were the commonest sources of referral as well as the number of health facilities in a region offering similar AA services [17].

The AA cases during the election year, 2017, were either at their highest or lowest levels in the study centers. The increase could have been due to the increased demand for AA certificates prior to registration for the national identity and electoral voter´s cards as has been previously observed by Kenya National Commission on Human Rights [18]. However, sources of referral during that period were not documented, hence, not verifiable. The decline in AA services may have followed a general decline in dental visits during the election year (2017) probably due to associated political instability [19]. Notably, dental visits increased in 2018 following peaceful national elections.

In most study centers, the frequency for AA was less than 10% among the dental patients as opposed to the North Eastern health facility which recorded a higher (38%) demand. Notably, such high numbers may lead to compromised AA procedures which are usually time-consuming especially if psychosocial history and medical methods are simultaneously applied. This may as well expose the already constrained dental clinicians and facilities to undue pressure, hence, delaying the prompt delivery of other urgent dental services. In such cases, hospitals may need to collaborate with other cadres of staff such as social workers and psychologists to carry out non-medical AA services, similar to the practice in the United Kingdom [11,20]. Referrals to the dental clinicians can then be undertaken when the age is disputed and dental age may provide additional information.

The five-year data showed that both females and males equally sought AA services. However, studies done in France and Denmark indicated minimal female involvement while males dominated at 98% and 93% respectively [12,13]. The differences could be due to varying reasons for AA. A review of the sampled population revealed that AA procedures are predominantly done on children. According to the Kenyan constitution, age determination plays a unique role in categorizing children under a specific age limit of either 8, 12, or 18 years [7]. Similar observations have been noted in other countries where most AA procedures are done on children or young adults in order to determine whether one has attained the country-specific legal age of majority. In Europe, this mostly involves young refugees, immigrants, or asylum seekers [11,13]. However, our study found that AA procedures were mostly sought prior to court proceedings or obtaining identification documents. Hence, the need for each country to customize its guidelines to address their unique needs.

According to the Kenya child protection data (2019/2020), children endure various challenges that may require court proceedings and confirmation of age. The top cases have been found to involve neglect (52.6%), custody (17.3%), abandonment (5.6%), defilement (4.1%), orphaned child (2.9%), parental child abduction (2.2%), physical abuse/violence (1.9%), lost and found children (1.6%), abduction (1.4%), child offender (1.3%) and registration (1.2%). About 4,024 (4.2%) children were referred to court for intervention [21]. Cases of defilement with disputed age are not uncommon in our setup. Victims may want to prove to be underage in order to seek compensation while the crime perpetrators may claim that their victim is of age in order to attract a less severe verdict [5,6,22]. Coincidentally, the data from the sampled population was collected when the schools were closed and the country was under lockdown due to the spread of the coronavirus disease of 2019 [23]. Therefore, a sampled population may have captured more children than usual since during that period there was an increase in violation of children´s rights in regard to neglect and defilement among others. Gender-based violence increased by 92.2% from January to June 2020 when compared with January to December 2019 and affected mostly (71%) females [24]. This explains the high number of females who sought AA services in 2019-2020 at the Rift Valley health facility.

Further sources of referral for AA were found to be associated with applications for registration, travel, or birth documents from the Department of Civil Registration, Immigration, and National Registration Bureau respectively. The services are provided by the Ministry of Interior and Co-ordination of National Government. Applicants are usually required to provide proof of their age and positive identification by local national government administration officers while the border and cosmopolitan applicants are vetted by identification committees which may refer for AA [15]. Majority of referrals from the North Eastern health facility were mostly for registration/identification documents. A previous study revealed that strict measures were applied while issuing identity cards in this region due to the difficulties associated with distinguishing between Somalis of Kenyan origin from those of Somalia. Notably, the area has high numbers of refugees and asylum seekers [25,26].

**Meaning of the study:** requests for AA will remain in demand; hence, relevant academic institutions and professional associations should embrace forensic dentistry. The dental curriculum should include a substantial amount of time dedicated to both theory and practical training in AA. There is also a need for practicing dentists to hone their skills and to be updated in the application of AA procedures. The information on sources of referral revealed that dentists have a social responsibility in providing justice to the community. The data also provide important baseline information on major stakeholders who can be involved in setting up guidelines for AA. It majorly includes the judiciary, hence, the need to bridge the gap between dentistry and justice through training dentists as expert witnesses.

**Unanswered questions and future research:** it was not investigated how much time was consumed while assessing the age of each patient. Hence, the implication of having a third of dental patients seeking AA in a day can only be assumed to be a challenge vis-a-vis attending to all other dental patients. We did not review the private sector or non-governmental organizations which could probably be having a different approach to AA. Further, a follow-up on the utility and implication of a given age to individuals should be undertaken so as to evaluate its effectiveness and validate the procedures. Hence, a training needs assessment in forensic dentistry is recommended.

**Limitations:** the generalization of the results is only limited to the public health hospitals. The results are only limited to AA in living individuals who were documented at the study centers. Information on the number of dental visits for AA was retrieved from manual records of which could be subject to counting errors. Reasons for AA over the period of 5 years could not be studied due to lack of reliable documentation.

## Conclusion

The 5-year retrospective review of selected level 5 hospitals across Kenya revealed that an average of 5% of dental patients visit for AA. Therefore, continued capacity building in AA in tertiary learning institutions and continuous professional development should be supported. In addition, a review of a sample of 316 AA patients revealed that the majority were referrals from the court of law. Hence, further research on how the dental fraternity can collaborate with the Judiciary to ensure fairness in age disputes is recommended.

### What is known about this topic

- *The age can be estimated by dentists through the observation of developmental and recessive features of teeth;*
- *Age assessment can be indicated for various reasons including social, civil, medical, forensics, and archeological purposes*.

### What this study adds

- *The actual proportions of individuals seeking AA services from selected public hospitals have been highlighted;*
- *The purpose of AA is mainly for investigative court proceedings and prior to application for civil registration documents*.
